# Hirudins and fenestrins of the African medicinal leech *Asiaticobdella fenestrata*

**DOI:** 10.1007/s00436-025-08578-x

**Published:** 2025-11-07

**Authors:** Lucia Schulz, Céline Tolksdorf, Bernhard H. Rauch, Sebastian Kvist, Christian Müller

**Affiliations:** 1 Animal Physiology, Zoological Institute and Museum, University of Greifswald, 17489 Greifswald, Germany; 2 Pharmacology and Toxicology, University Medicine Oldenburg, Carl Von Ossietzky University Oldenburg, Oldenburg, Germany; 3 Research Division, Swedish Museum of Natural History, 104 05 Stockholm, Sweden

**Keywords:** Hirudin, Fenestrin, Blood coagulation, Platelet aggregation, Hematophagous leeches

## Abstract

African hematophagous leeches are largely underinvestigated both in terms of their biodiversity and the characterization of the repertoire of bioactive components of their salivary gland secretions. *Asiaticobdella* (formerly *Aliolimnatis*) *fenestrata* Moore, 1939, also referred to as the African medicinal leech, is hence a promising candidate for the identification and functional characterization of putative anticoagulants that may serve as inspirations for the development of new or the enhancement of existing drugs for medical purposes. *A. fenestrata* inhabits flooded plains of southern African regions and mainly feeds on mammals like black lechwe (*Kobus leche* Gray, 1850). Whereas several anticoagulants, including two putative hirudins of *A. fenestrata*, have already been identified in previous investigations, functional characterizations of these components are yet missing. We conducted an in-depth re-analysis of the salivary gland transcriptomic data set of *A. fenestrata* and identified two additional hirudin-like factors that exhibit a remarkable structural feature—the presence of an RGD motif at or near the N-terminal end of the molecules. The presence of such an RGD motif is a key feature of leech-derived platelet aggregation inhibitors such as ornatins and decorsins. However, both in ornatins and in decorsins, the respective RGD motif is located within the molecule between the 5th and the 6th cysteine residues, but not at or near the N-terminus. We named the two putative platelet aggregation inhibitors “fenestrins.” Both the two putative hirudins and the two fenestrins of *A. fenestrata* were expressed as recombinant proteins, purified, and functionally characterized in either coagulation or platelet aggregation assays.

**Supplementary Information:**

The online version contains supplementary material available at 10.1007/s00436-025-08578-x.

## Introduction

The indigenous African medicinal leeches *Asiaticobdella fenestrata* Moore, 1939, and *A. buntonensis* Meyer, 1951, are jawed leeches that belong to the family Hirudinidae (Phillips and Siddall 2009; Siddall et al. 2011). They inhabit flooded plains of southern Africa, are hematophagous, and mainly feed on fishes or larger animals like hippopotamus or black lechwe (Oosthuizen 1991; Kvist et al. 2013). Despite their great potential for medical applications (Wilken and Appleton 1993), the exact composition of their salivary gland secretions remains largely unexplored. So far, only one study specifically addressed the investigation of the transcriptome profile of the salivary glands of *A. fenestrata*, and the expression of several putative anticoagulants, including two hirudins, could be determined (Kvist et al. 2013; Müller et al. 2019). However, functional characterizations of all putative anticoagulants are still missing.

In previous studies, we have already successfully applied the strategy to re-investigate existing transcriptomic and/or genomic data sets for the presence of putative anticoagulants or other “proteins of interest” (Müller et al. 2022, 2024; Pfordt et al. 2022). Hence, we conducted a similar analysis of the salivary gland transcriptomic data set of *A. fenestrata* (Kvist et al. 2013) with special emphasis on the identification of members of the hirudin superfamily. Hirudins are by far the most widely explored leech-derived anticoagulants (Markwardt 1993). The archetype hirudin of the European medicinal leech *Hirudo medicinalis* Linne, 1758, is a 65 amino acid polypeptide (w/o the signal peptide sequence) that comprises a molecular mass of about 7 kDa and an acidic isoelectric point (pI) of about 4.2 (Dodt et al. 1984). The structure of all hirudins is characterized by six conserved cysteine residues and the presence of three functionally distinct domains: a short N-terminal sequence of five amino acid residues that blocks the active site of thrombin, a central globular domain that is stabilized by three disulfide bonds, and an elongated C-terminal tail that blocks the fibrinogen binding site of thrombin (Rydel et al. 1991). Hirudins are therefore known as bivalent thrombin inhibitors (Warkentin 2004). Hirudin-like factors (HLFs) share certain structural (e.g., the presence and arrangement of the six cysteine residues within the central globular domain) and genomic (a conserved gene structure that is comprised of four exons and three introns) features with hirudins (Müller et al. 2016). However, both factors may significantly differ in other aspects, such as molecular masses and isoelectric points (pI values) (Müller et al. 2017). While certain HLFs display thrombin-inhibitory potencies equivalent to those of hirudins, others demonstrate either minimal or no measurable thrombin inhibition (Müller et al. 2016, 2020a; Pfordt et al. 2022). The first aim of the present study was hence to test whether or not the putative hirudins of *A. fenestrata* are indeed strong thrombin inhibitors.

Beside hirudins and HLFs, decorsins of *Macrobdella decora* Say, 1824 (Seymour et al. 1990), and ornatins of *Placobdella ornata* Verrill, 1872 (Mazur et al. 1991), are members of the hirudin superfamily too. Both factors are inhibitors of platelet aggregation, and the inhibition is mediated via an RGD motif that is located on the tip of a loop between the 5th and the 6th cysteine residues of the central globular domain (de Laureto et al. 1998) and binds to glycoprotein IIb/IIIa (GPIIb/IIIa) on platelets (Mazur et al. 1991). The presence of an RGD motif is a hallmark of GPIIb/IIIa antagonists (Lazarus and McDowell 1993; Reiss et al. 2006). Strikingly, the re-analysis of the salivary gland transcriptomic data set of *A. fenestrata* revealed the presence of cDNAs for two HLFs that comprise RGD motifs at or near the N-terminal end of the respective proteins. The presence of an RGD motif within the C-terminal tail of certain haemadins (hirudin-like thrombin inhibitors of Asian land leeches of the genus *Haemadipsa* Tennent, 1859) did not facilitate inhibition of platelet aggregation (Müller et al. 2024). Therefore, the second aim of the present study was to evaluate whether or not the presence of an RGD motif at or near the N-terminus of a HLF facilitates the inhibition of platelet aggregation.

## Materials and methods

### Transcriptomic data

Transcriptomic data of *Asiaticobdella fenestrata* were obtained from the GenBank EST database (accession numbers JZ183761–JZ185315, Kvist et al. 2013).

### Bioinformatic and graphical tools

Basic Local Alignment Search Tool (BLAST) searches were performed using either the NCBI web portal (https://blast.ncbi.nlm.nih.gov/Blast.cgi) or BioEdit v7.2.5 (Hall 1999) and adjusted parameter settings for both word size and the expected threshold values. Multiple sequence alignment (MSA) files were generated using either ClustalX 2.1 (Larkin et al. 2007) or the CLC Sequence Viewer software package v8.0 (QIAGEN, Aarhus, Denmark) at default settings. Respective alignments were exported as msf-files and further processed using Gene-Doc v2.7 (Nicholas and Nicholas 1997). Signal peptide sequences were predicted using the Phobius web portal (Käll et al. 2007) and SignalP6.0 (Teufel et al. 2022). Graphs were generated and analyzed using GraphPad Prism V5.01 (GraphPad Software, Boston, MA, USA). 

### Gene synthesis

cDNA fragments of putative hirudins and HLFs were generated using the GeneArt gene synthesis service of ThermoFisher Scientific (Darmstadt, Germany).

### Amplification and cloning of putative hirudin and fenestrin cDNAs

For the amplification of putative hirudins and fenestrins cDNAs, primers were derived from the respective transcriptomic sequences. A list of all primers that were used in the study is provided in Supplementary Information Table S1. PCR reactions were performed using Q5 high-fidelity DNA polymerase (New England Biolabs, Frankfurt a. M., Germany); fragments of relevant sizes were purified and cloned into the expression vector pQE30Xa (QIAGEN, Hilden, Germany). Successfully cloned cDNAs were sequenced for control purposes by Biosearch Technologies (LGC, Berlin, Germany). 

### Expression, purification, processing, and quantification of putative hirudins and fenestrins

The detailed protocol to express, purify, process, and quantify the respective recombinant proteins was described in numerous recent publications (Müller et al. 2016, 2020a, 2020b; Pfordt et al. 2022; Wang et al. 2025). Briefly, all factors were expressed in the widely used laboratory strain *Escherichia coli* DH5α (Hanahan 1985), and we applied an expression and purification system that was developed by QIAGEN (Hilden, Germany). The pQE30Xa vector encodes a factor Xa protease recognition site that is located between the His-tag coding region at the 5′ side and the multiple cloning site at the 3′ side. A subsequent factor Xa protease treatment hence cleaves off the His-tag and results in a recombinant protein that is devoid of any vector-derived amino acid residues at the N-terminus. Molar concentrations of final protein solutions were calculated by dividing the absorbance at 280 nm by the molar absorption coefficient according to the equation ε = (nW × 5,500) + (nY × 1,490) + (nC × 125) (Gill and von Hippel 1989; Pace et al. 1995). Gel images after the His-Tag purification steps and the final concentrations of all factors are provided in Supplementary Information Figure S1 and Table S2, respectively.

### Blood coagulation assays

To verify the thrombin-inhibitory potencies of putative hirudins and fenestrins of *A. fenestrata*, we performed the thrombin time test (TTT; reference range 16.8–21.4 s) using a BFT II analyzer (Siemens Healthcare, Erlangen, Germany). All steps were carried out according to the manufacturer’s instructions. Protein samples were diluted with buffer to reach final concentrations in the reaction assays of 3.2 μmol/l, 0.32 μmol/l, or 0.032 μmol/l, respectively. The desired amount of substrate was directly transferred into the test cuvette immediately before the plasma was added. Dade® Ci-Trol® 1 (Siemens Healthcare, Erlangen, Germany) was used as standardized human plasma. The incubation of reaction mixtures was carried out at 37.4 °C. Measurements that reached 300 s before any coagulation was detected were stopped and considered as complete inhibition of coagulation. Blood coagulation tests were performed in three technical replicates.

### Platelet aggregation assays

All assays were performed with human blood samples that were obtained from healthy human volunteers after written informed consent and approval from the institutional ethics committee. Blood collection, sample preparation, and the subsequent experimental procedure were performed as described in Müller et al. (2024). Briefly, 10 ml of venous blood was taken from the antecubital vein using an S-Monovette® (Sarstedt, Nürnbrecht, Germany) prefilled with citrate buffer. The first centrifugation step of the blood collection tube was performed at 200 g for 20 min. After centrifugation, the supernatant (platelet-rich plasma, PRP) was transferred, and the remaining blood was centrifuged again for 10 min at 2000 g and room temperature. The supernatant was dedicated as platelet-poor plasma (PPP), transferred, and used as a reference value for maximal platelet aggregation. Measurements were performed using a TA-8 V aggregometer (Diagnostica Stago S.A.S., Asnières-sur-Seine, France). The snake venom-derived platelet aggregation inhibitors tirofiban and eptifibatide (Sigma-Aldrich, Taufkirchen, Germany) were used as positive controls for complete inhibition of platelet aggregation. PRP was pre-incubated with the respective test and control compounds (final concentration 3.2 μmol/l) or buffer for 1 min at 37 °C. For the measurement, PRP was then transferred into test cuvettes and stimulated with ADP (200 μmol/l; Hart Biologicals, Hartlepool, UK; final concentration 5 μmol/l) after 1 min of runtime. The final volume in each test cuvette was 250 μL of diluted PRP. All experiments were performed at 37 °C over a time period of 400 s. Maximal aggregation in percentage and the area under the curve were calculated as quantitative output parameters (Zhou and Schmaier 2005). Platelet aggregation tests were performed in two to four technical replicates.

## Results

### Identification of putative hirudins and fenestrins in *A. fenestrata*

**Fig. 1:**
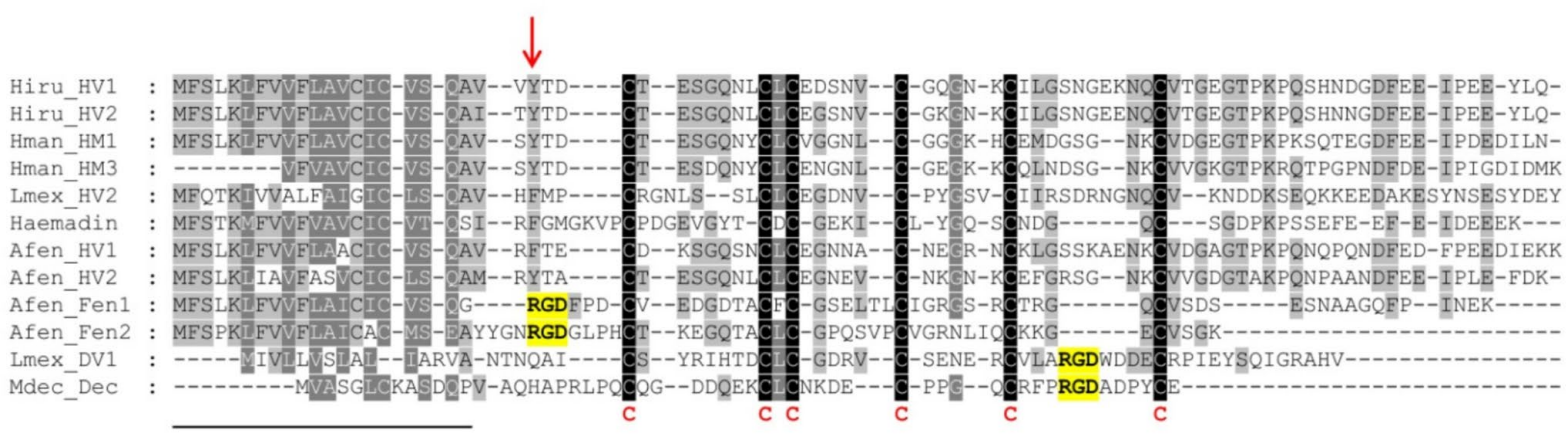
Multiple amino acid sequence alignments of hirudin variants HV1 and HV2 of *H. medicinalis* (Hmed_HV1 and Hmed_HV2); HM1 and HM3 of *Hirudinaria manillensis* Lesson, 1842 (Hman_HM1, Hman_HM3); HV2 of *Limnobdella mexicana* Blanchard, 1893 (Lmex_HV2); haemadin of *Haemadipsa sylvestris* Blanchard, 1894, decorsins of *M. decora* (Mdec_Dec) and *L. mexicana* (Lmex_DV1); and the putative hirudins (Afen_HV1 and Afen_HV2) and fenestrins (Afen_Fen1 and Afen_Fen2) of *A. fenestrata*. A black background indicates fully conserved residues; a gray background indicates partially conserved residues. The six conserved cysteine residues giving rise to the three-dimensional structure of the central globular domain are marked in bold and red. The RGD motifs are marked in bold and yellow. The tyrosine or phenylalanine residue at position 3 is marked with an arrow. The signal peptide sequence is underlined. Abbreviations are used according to the IUPAC code

Previous investigations revealed the presence of two putative hirudins in transcriptomic data of salivary gland cell preparations of *A. fenestrata*, namely Afen_HV1 (GenBank EST JZ185150; Kvist et al. 2013) and Afen_HV2 (GenBank EST JZ184165; Müller et al. 2019). Both putative hirudins exhibit typical structural (see Fig. 1) and biochemical (see Table 1) features of hirudins. The structural features comprise a length of about 65 amino acids, the presence and distribution pattern of six cysteine residues, a short N-terminus with a tyrosine or phenylalanine residue at position 3, and an elongated acidic C-terminal tail. The biochemical features comprise an isoelectric point value (pI) of about 4–5 and a molecular mass of about 7 kDa.

**Table 1:**
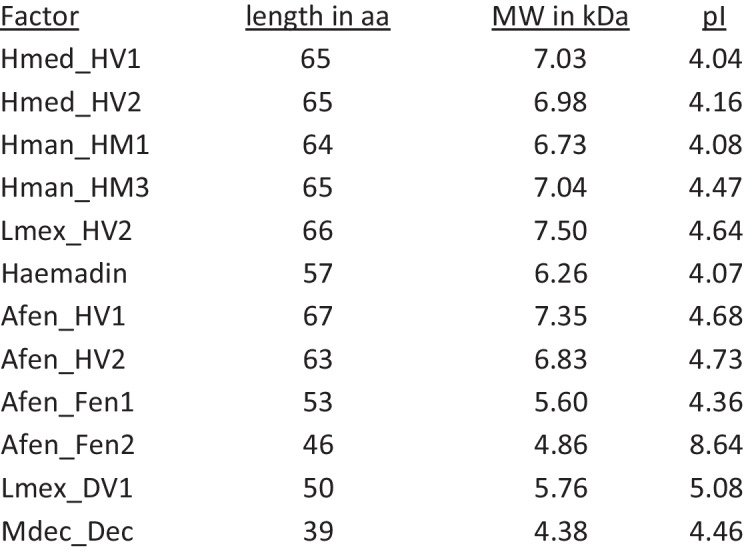
Structural and biochemical features of hirudin variants HV1 and HV2 of *H. medicinalis* (Hmed_HV1 and Hmed_HV2), HM1 and HM3 of *H. manillensis* (Hman_HM1, Hman_HM3), HV2 of *L. mexicana* (Lmex_HV2), haemadin of *H. sylvestris*, decorsins of *M. decora* (Mdec_Dec) and *L. mexicana* (Lmex_DV1), and the putative hirudins (Afen_HV1 and Afen_HV2) and fenestrins (Afen_Fen1 and Afen_Fen2) of *A. fenestrata*. The length is without the signal peptide sequence. *aa*, amino acids; *MW*, molecular weight; *kDa*, kilodalton; *pI*, isoelectric point

An in-depth re-analysis of the salivary gland EST data set of *A. fenestrata* revealed strong evidence for the presence of two additional sequences that encode for members of the hirudin superfamily, namely Afen_Fen1 (GenBank EST JZ185006) and Afen_Fen2 (GenBank EST JZ185191). The complete amino acid sequences of both factors are provided in Fig. 1; the relevant biochemical features are listed in Table 1. Both factors are shorter compared to hirudins and in the size range of decorsins, but comprise the canonical pattern of six cysteine residues within the central globular domain (see Fig. 1). Whereas the pI value of Afen_Fen1 is acidic (4.36) and matches the pI values of hirudins, the pI value of Afen_Fen2 is basic (8.64) (see Table 1). Strikingly, both factors comprise an RGD motif at (Afen_Fen1) or near (Afen_Fen2) the N-terminus (see Fig. 1). As a consequence, they lack the conserved tyrosine or phenylalanine residues at position 3, a hallmark of hirudin’s inhibitory effect on thrombin (Lazar et al. 1991; Huang et al. 2014). In contrast, the RGD motif is pivotal for the inhibition of platelet aggregation by decorsin (Krezel et al. 1994), but is located between the 5th and the 6th cysteine residue (see Fig. 1). Taken together, Afen_Fen1 and Afen_Fen2 may represent a new type of leech-derived factors of the hirudin superfamily and were hence termed fenestrins. The cDNA and the deduced amino acid sequences of both the putative hirudins and the fenestrins of *A. fenestrata* are provided in Supplementary Information Figure S1.

## Functional characterization I: thrombin inhibition

**Fig. 2:**
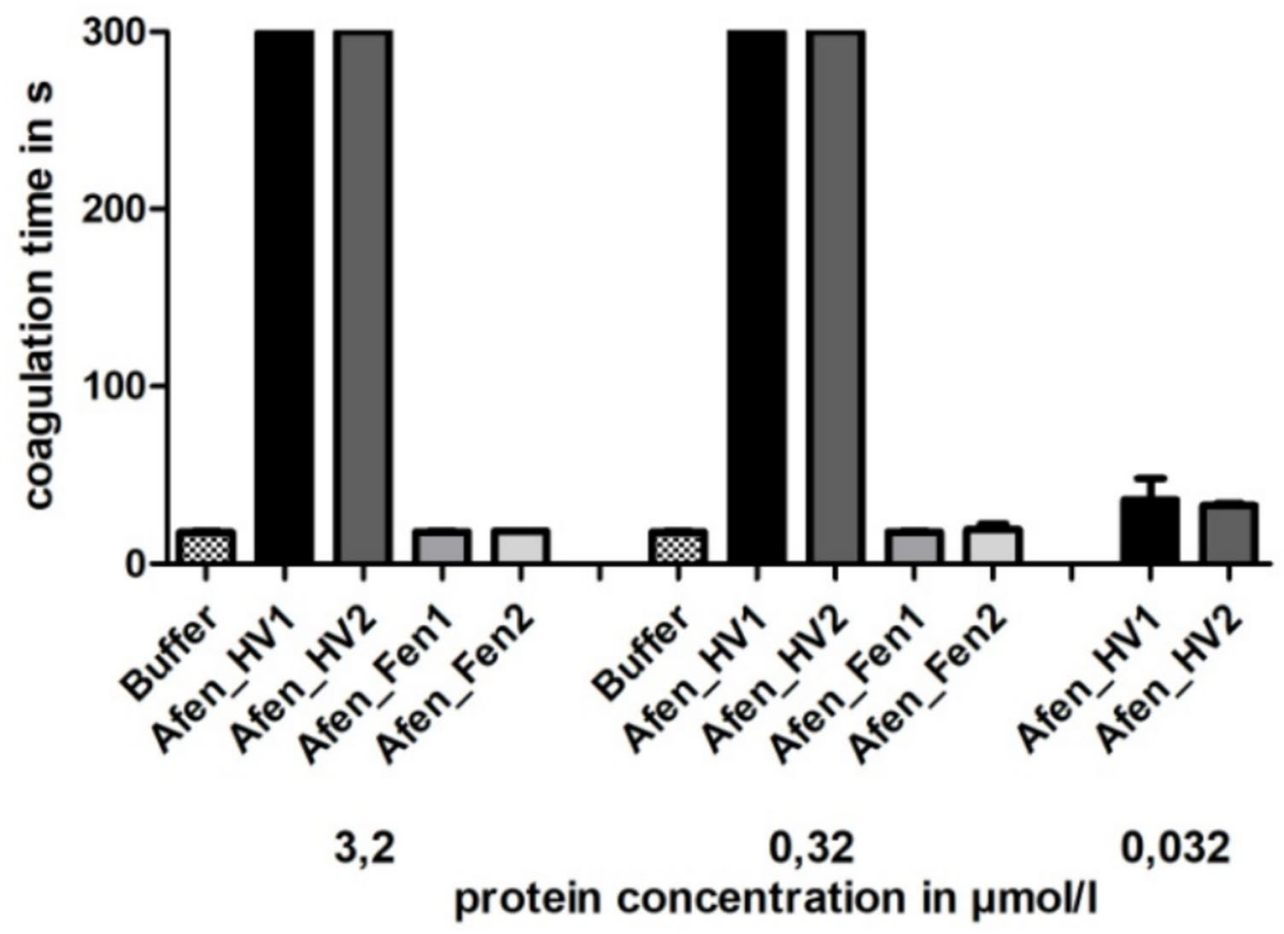
Standard blood coagulation assays of putative hirudins (Afen_HV1 and _HV2) and fenestrins (Afen_Fen1 and Afen_Fen2) of *A. fenestrata* using the thrombin time test (TT). Results are the mean of three independent measurements

Both the two putative hirudins and the two fenestrins of *A. fenestrata* were functionally characterized in terms of their potencies to inhibit the activity of thrombin and hence block the coagulation cascade. For that, all factors were successfully expressed, purified, processed, and tested in the thrombin time test coagulation assay. Whereas both putative hirudins of *A. fenestrata* are very strong thrombin inhibitors, neither Afen_Fen1 nor Afen_Fen2 displayed any thrombin-inhibitory potency at the tested concentrations (Fig. 2).

### Functional characterization II: platelet aggregation

**Fig. 3:**
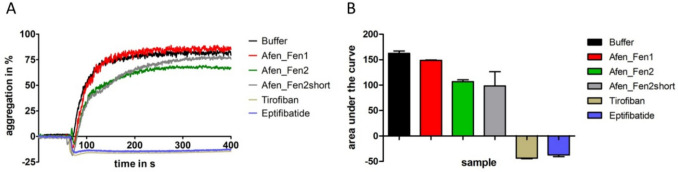
Platelet aggregation determined by light transmission in response to ADP in the absence or presence of the fenestrin variants Afen_Fen1, _Fen2, and _Fen2short of *A. fenestrata*. **A** Platelet aggregation curves. **B** Area under the curve values. Tirofiban and eptifibatide were used as positive control compounds for the complete inhibition of aggregation; buffer was used as a negative control. All factors and the control compounds were tested at a final concentration of 3.2 μmol/l. Platelet aggregation was induced by the addition of ADP to a final concentration of 5 μmol/l. Results are the mean of two to four independent measurements

To determine the inhibition of ADP-induced platelet aggregation, only the fenestrins of *A. fenestrata* were tested. Afen_Fen2 was used either unmodified (Afen_Fen2) or in an N-terminally truncated version (Afen_Fen2short) that lacked the first four amino acid residues of Afen_Fen2 (YYGN, see Fig. 1) to locate the RGD motif of Afen_Fen2short directly at the N-terminus. The snake venom-derived platelet aggregation inhibitors tirofiban and eptifibatide were used as positive controls. As shown in Fig. 3A, none of the fenestrins of *A. fenestrata* inhibited the platelet aggregation to an extent comparable to the (maximally inhibitory) positive controls. However, the second wave of platelet aggregation induced by ADP was attenuated by Afen_Fen2 and Afen_Fen2short, respectively, resulting in a slight reduction in both the maximum platelet aggregation for Afen_F2a (Fig. 3A) and the area under the curves for both fenestrin variants Afen_Fen2 and Afen_Fen2short (Fig. 3B).

## Discussion

African hematophagous leeches are an integral part of the local fauna, and their use for medical purposes traces back to ancient Egypt (Whitaker et al. 2004). However, they are largely under-represented in terms of the identification and functional characterization of bioactive components of their salivary gland secretions. Only very recently was a first article on the diversity of hirudin and HLF genes in the North-African medicinal leech, *Hirudo troctina* Johnson, 1816, including a functional characterization of the respective factors, published (Ben Ahmed et al. 2024). Whereas *H. troctina* belongs to the genus *Hirudo* that inhabits the Western Palaearctic region (Utevsky et al. 2010), *A. fenestrata* and its sister species *A. buntonensis* are indigenous to southern Africa (Oosthuizen and Curtis 1990; Oosthuizen 1991; Wilken and Appleton 1993). Kvist et al. (2013) generated and analyzed a salivary gland transcriptome of *A. fenestrata* and identified several putative anticoagulants, including bdellins, antistasins, and one hirudin, but did not perform any functional characterizations. Here, we describe the recombinant expression, purification, and functional characterization of two putative hirudins of *A. fenestra*. In addition, we identified two factors that resemble HLFs but contain an RGD motif at or near their N-terminal end and may hence comprise platelet-aggregation inhibitory potencies.

Both putative hirudins of *A. fenestrata* exhibited very high thrombin-inhibitory potencies (see Fig. 2) comparable to the hirudins of *H. medicinalis* (Müller et al. 2020a), *L. mexicana* (Pfordt et al. 2022), and *H. troctina* (Ben Ahmed et al. 2024). Interestingly, Afen_HV1 comprises a phenylalanine residue at position 3 (Phe3), like the haemadin of *H. sylvestris* (Strube et al. 1993), or the hirudins of *L. mexicana* (Pfordt et al. 2022). In contrast, Afen_HV2 contains the more common Tyr3 residue instead (see Fig. 1). In the hirudin variant HV1 of *H. medicinalis*, replacing Tyr3 with Phe3 increases its affinity to thrombin by about sixfold (Lazar et al. 1991); conversely, in haemadin from *H. sylvestris*, replacing Phe3 with Tyr3 reduces its affinity to thrombin by a factor of 3.5 (Acquasaliente et al. 2023). Nevertheless, both hirudins of *A. fenestrata* exhibit almost the same thrombin-inhibitory potencies (see Fig. 2). Hence, it might hence be possible that the putative N-terminus-mediated higher affinity of Afen_HV1 to thrombin is compensated by other structural features in Afen_HV2, e.g., the slightly more acidic pI value of its C-terminal tail (4.44 in Afen_HV2 compared to 4.61 in Afen_HV1) in combination with the presence of a canonical DFxxIP motif (DFxxEP in Afen_HV1, see Fig. 1). In either case, both hirudins of *A. fenestrata* are promising candidates for the development of highly effective alternatives to the established hirudin/bivalirudin for therapeutical applications and clinical use.

Hirudins as thrombin inhibitors address the secondary hemostasis (the blood coagulation), whereas inhibitors of platelet aggregation address the primary hemostasis. Respective inhibitors of platelet aggregation are calin and saratin of *H. medicinalis* (Munro et al. 1991; Barnes et al. 2001). Both inhibitors block the von Willebrand factor-mediated binding of platelets to collagen (Harsfalvi et al. 1995; Barnes et al. 2001). The molecular identity of calin still remains obscure, but the coding sequence of a putative saratin was identified in *A. fenestrata* (Kvist et al. 2013; GenBank JZ184186.1). In contrast to calin and saratin, the inhibitors decorsin of *M. decora* (Seymour et al. 1990) and ornatin of *P. ornata* (Mazur et al. 1991) are potent GPIIb/IIIa-inhibitors and hence block the interaction between platelets and fibrinogen. Both factors belong to the hirudin superfamily. The interaction between decorsin/ornatin and GPIIb/IIIa is mediated via an RGD motif that is located between the 5th and the 6th cysteine residues (marked in yellow in Fig. 1) (de Laureto et al. 1998). Platelet aggregation inhibitors of the decorsin/ornatin type have been identified and functionally characterized in the American leeches *L. mexicana* and *H. vizottoi* (Pfordt et al. 2022) and in the Asian land leech *Haemadipsa interrupta* Moore, 1835 (Müller et al. 2024), but are apparently absent in *H. medicinalis* and its closest European relatives. Our re-investigations of the salivary gland cells transcriptome data set of *A. fenestrata* revealed the expression of two factors, namely Afen_HLF1 (or fenestrin Afen_Fen1) and Afen_HLF2 (or fenestrin Afen_Fen2), that also belong to the hirudin superfamily but lack typical structural and biochemical features of hirudins (see Fig. 1 and Table 1). In fact, both fenestrins do not inhibit the activity of thrombin (see Fig. 2). But strikingly, both factors comprise an RGD motif that is, however, located at or near the N-terminus (marked in yellow in Fig. 1). We have hence functionally characterized the fenestrins of *A. fenestrata* to evaluate their platelet aggregation-inhibitory potencies. Our results indicate that Afen_Fen1 does not inhibit platelet aggregation, whereas Afen_Fen2 derivatives comprise weak but detectable platelet aggregation-inhibitory potencies (see Fig. 3). Notably, the second wave of aggregation is attenuated. This phase is typically mediated by the release of ADP or thromboxane A2 (TXA_2_) from the granules of activated platelets. Inhibition of these pathways (e.g., by blockade of P2Y_1_/P2Y_12_ or cyclooxygenase inhibition) can result in a reduced slope of the second aggregation curve (Dawood et al. 2007). Whether fenestrins inhibit platelet aggregation in part through blockage of specific receptors involved in granule release, or via different mechanisms, remains to be elucidated. These effects, however, were considerably less pronounced in comparison to both the positive controls eptifibatide and tirofiban and to the decorsins of *L. mexicana* and *H. vizottoi* (Pfordt et al. 2022). Nevertheless, the expression of respective cDNAs indicates that the corresponding fenestrin genes are active and that the factors are hence of biological importance for *A. fenestrata*. It might be that both fenestrins either address targets that are entirely different from platelet aggregation or that they require yet unknown co-factors to exhibit their full inhibitory potencies on platelet aggregation. It remains to be clarified to what extent the putative saratin of *A. fenestrata* actually inhibits platelet aggregation.

The availability of high-quality chromosome-level genomic data of hematophagous leeches increased over the last years, but is still rather limited. Whenever performed, precise annotations revealed the presence of numerous genes that encode putative anticoagulants, including several hirudins and hirudin-like factors (Liu et al. 2023, 2024; Zhao et al. 2024, 2025; Khan et al. 2025). To date, no genomic data of *A. fenestrata* are available. Despite the apparent lack of support by the transcriptomic data, it is, however, not implausible to assume that Afen_HV1/HV2 and Afen_Fen1/Fen2 may not represent the full repertoire of members of the hirudin superfamily in *A. fenestrata*. Hence, further investigations based on genomic data may not only allow for the identification and annotation of the genes for the factors that were analyzed in the present study, but may also lead to the discovery of new candidates for structural and functional characterizations. 

## Supplementary Information

Below is the link to the electronic supplementary material.

ESM 1 Supplementary Material 1 (PDF 814 KB)

## Data Availability

Original sequence data are deposited in the GenBank EST database (accession numbers JZ183761—JZ185315). Specific entry numbers are listed within the manuscript and the respective cDNA and deduced amino acid sequences are provided as Supplementary Information. Images and data that illustrate the expression, purification and processing of all recombinant factors are either provided as Supplementary Information or are available on request from the corresponding author.
