# Supplementary material for: Hirudins and fenestrins of the African medicinal leech Asiaticobdella fenestrata
Source: Parasitol Res. 2025 Nov 7;124(11):124. doi: 10.1007/s00436-025-08578-x (PMC12592272; doi:10.1007/s00436-025-08578-x)
Supplement: Supplementary file 1 — Supplementary Material 1 (PDF 814 KB) [file 436_2025_8578_MOESM1_ESM.pdf]

Figure S1

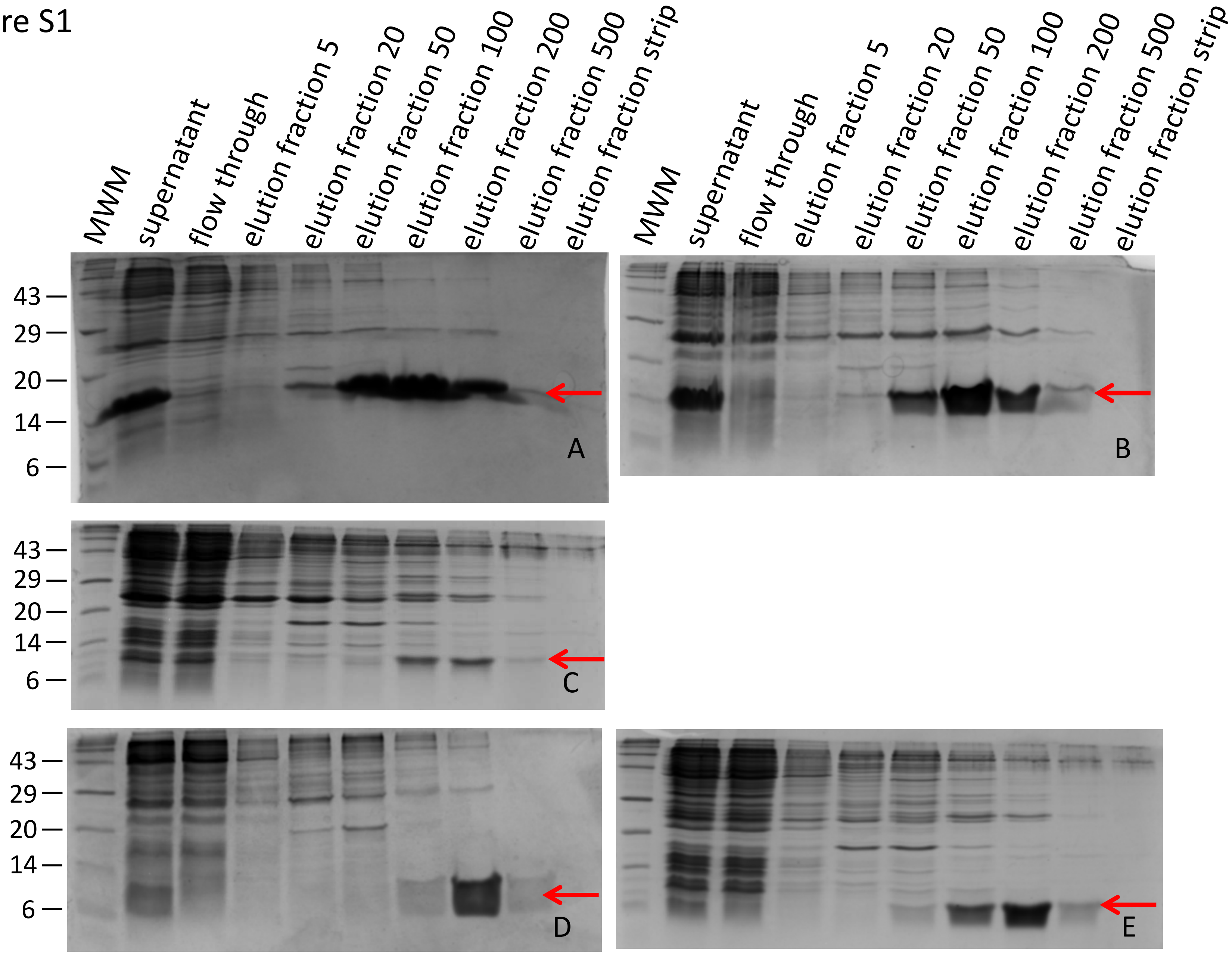
MWM
supernatant
flow through
elution fraction 5
elution fraction 20
elution fraction 50
elution fraction 100
elution fraction 200
elution fraction 500
elution fraction strip
43
29
20
14
6
A
B
C
D
E

Figure S2

JZ185150 = Afen_HV1

ATGTTCTCTCTTAAGTTGTTCGTTGTCTTCTTGGCTGCTTGCATCTGCGTGTCTCAAGCAGTGAGATT
CACTGAA**TGT**GATAAGTCAGGTCAAAGTAAT**TGC**CTT**TGT**GAAGGTAATAATGCG**TGC**AATGAAGGCC
GAAAT**TGT**AAATTGGGCAGTTCTAAAGCTGAAAATAAA**TGT**GTCGATGGAGCGGGTACTCCGAAGCCT
CAAAACCAGCCTCAAAACGATTTCGAAGATTTCCCCGAGGAAGATATTGAAAAGAAA**TAA**

MFSLKLFVVFLAACICVSQAVRFTE**C**DKSGQSN**C**L**C**EGNNA**C**NEGRN**C**KLGSSKAENK**C**VDGAGT**PKP**
QNQPQNDFEDFPEEDIEKK

VRFTE**C**DKSGQSN**C**L**C**EGNNA**C**NEGRN**C**KLGSSKAENK**C**VDGAGT**PKP**QNQPQNDFEDFPEEDIEKK

Theoretical pI/Mw: **4.68** / 7353.97

JZ184165 = Afen_HV2

ATGTTTTCTCTTAAATTAATCGCTGTTTTCGCGTCTGTTTGCATCTGTCTGTCTCAAGCCATGAGATA
CACTGCT**TGT**ACAGAGTCAGGTCAAAATCTA**TGT**CTC**TGC**GAGGGAAATGAAGTC**TGC**AATAAAGGCA
ATAAA**TGT**GAATTTGGCAGAAGTGGAAACAAA**TGT**GTTGTTGGGGATGGTACTGCGAAGCCTCAAAAC
CCGGCTGCAAACGACTTTGAAGAAATTCCTTTAGAGTTCGATAAA**TGA**

MFSLKLIAVFASVCICLSQAMRYTA**C**TESGQNL**C**L**C**EGNEV**C**NKGNK**C**EFGRSGNK**C**VVGDGT**AKP**QN
PAAND**F**EE**IP**LEFDK

MRYTA**C**TESGQNL**C**L**C**EGNEV**C**NKGNK**C**EFGRSGNK**C**VVGDGT**AKP**QNPAAND**F**EE**IP**LEFDK

Theoretical pI/Mw: **4.73** / 6832.59

JZ185006 = Afen_Fen1

ATGTTCTCTCTCAAGTTGTTCGTTGTCTTTTTGGCGATATGCATTTGCGTGTCTCAAGGC**AGGGGAGA**
TTTCCCAGAT**TGT**GTAGAAGATGGTGATACAGCA**TGT**TTT**TGT**GGGTCCGAGTTGACACTC**TGC**ATTG
GTCGTGGCAGTAGG**TGC**ACTAGAGGACAA**TGT**GTTTCTGACAGTGAATCTAACGCTGCTGGACAATTT
CCAATTAATGAAAAA**TGA**

MFSLKLFVVFLAICICVSQG**RGD**FPD**C**VEDGDTA**C**F**C**GSELTL**C**IGRGSR**C**TRGQ**C**VSDSESNAAGQF
PINEK

RGDFPD**C**VEDGDTA**C**F**C**GSELTL**C**IGRGSR**C**TRGQ**C**VSDSESNAAGQFPINEK

Theoretical pI/Mw: **4.36** / 5604.12

JZ185191 = Afen_Fen2

ATGTTCTCTCCCAAGTTGTTCGTTGTCTTTTTGGCGATCTGCGCCTGCATGTCTGAAGCATATTATGG
AAAT**AGAGGAGAT**GGTCTCCCACAT**TGT**ACAAAGGAGGGTCAAACAGCA**TGT**TTA**TGT**GGGCCCCAGT
CGGTACCT**TGC**GTTGGTAGAAATTTAATACAG**TGT**AAGAAAGGAGAA**TGT**GTTTCTGGCAAA**TGA**

MFSPKLFVVFLAICACMSEAYYGN**RGD**GLPH**C**TKEGQTA**C**L**C**GPQSVP**C**VGRNLIQ**C**KKGE**C**VSGK

YYGN**RGD**GLPH**C**TKEGQTA**C**L**C**GPQSVP**C**VGRNLIQ**C**KKGE**C**VSGK

Theoretical pI/Mw: **8.64** / 4856.58

Table S1 List of oligonucleotide primers used in the study

Afen_HV1_fw:	5`-GTGAGATTCACTGAATGTGATAAG-3`
Afen_HV1_rev:	5`-TTAAGCTTATTTCTTTTCAATATCTTCC-3`
Afen_HV2_fw:	5`-ATGAGATACACTGCTTGTACAGAG-3`
Afen_HV2_rev:	5`-TTAAGCTTCATTTATCGAACTCTAAAGG-3`
Afen_HLF1_fw:	5`-AGGGGAGATTTCCCAGATTGTGTAG-3`
Afen_HLF1_rev:	5`-TTAAGCTTCATTTTTCATTAATTGGAAATTC-3`
Afen_HLF2_fw1:	5`-TATTATGGAAATAGAGGAGATGG-3`
Afen_HLF2_fw2:	5`-AGAGGAGATGGTCTCCCACATTGTAC-3`
Afen_HLF2_rev:	5`-TTAAGCTTCATTTGCCAGAAACACATTC-3`

Table S2 Concentrations of purified and processed recombinant hirudins Afen_HV1 and Afen_HV2 and recombinant fenestrins Afen_Fen1, AfenFen2 and Afen_Fn2short, respectively, after expression in *E. coli* strain DH5α.

Factor	Concentrations
Afen_HV1	286.7 µmol/l (2108 µg/ml)
Afen_HV2	204.9 µmol/l (1400 µg/ml)
Afen_Fen1	102.7 µmol/l (575 µg/ml)
Afen_Fen2	47.3 µmol/l (222 µg/ml)
Afen_Fen2short	182.7 µmol/l (797 µg/ml)
